# Knowledge, attitude and practice of students towards blood donation in Arsi university and Adama science and technology university: a comparative cross sectional study

**DOI:** 10.1186/s12878-017-0092-x

**Published:** 2017-11-21

**Authors:** Habtom Woldeab Gebresilase, Robera Olana Fite, Sileshi Garoma Abeya

**Affiliations:** 1 Department of public Health, College of Health Sciences, Adama General Hospital and Medical College, Adama, Ethiopia; 2 Department of Nursing, College of Health Sciences and Medicine, Woliata Sodo University, PO-Box: 138, Woliata sodo, Ethiopia; 3 Department of Social and Population Health, Adama General Hospital and Medical College, Adama, Ethiopia

**Keywords:** Knowledge, Practice, Attitude, Health science, Non-health science

## Abstract

**Background:**

Blood can save millions of lives. Even though people do not donate blood regularly, there is a constant effort to balance the supply and demand of blood. The aim of this study was, therefore, to determine the knowledge, attitude and practice of blood donation between university students.

**Methods:**

The comparative cross sectional study design was used in Adama Science and Technology University and Arsi University from April 11–May 2, 2016.360 students were selected using stratified sampling. Frequencies and proportions were computed. Chi-Square and logistic regressions were carried out and associations were considered significant at *p*<0.05.

**Result:**

The study revealed that there was a significant knowledge difference (χ2 = 152.779, *p*<0.001) and Attitude difference (χ2 = 4.142, *p* = 0.042) between Health Science students of Arsi University and Non-Health Science students of Adama Science and Technology University. The gender of the students (AOR = 3.150, 95% CI: 1.313, 7.554) was a significant predictor of the level of knowledge of Health Science students. The ethnicity of students (AOR = 2.085, 95% CI: 1.025, 4.243) was a significant predictor of the level of an attitude of Health Science students and gender of students (AOR = 0.343, 95% CI: 0.151, 0.779) was a significant predictor of the level of an attitude of Health Science students. Concerning Non-Health Science students, religion (AOR = 10.173, 95% CI: 1.191, 86.905) and original residence (AOR = 0.289, 95% CI: 0.094, 0.891) were a significant predictor of the level of knowledge of Non-Health Science students. Gender (AOR = 0.389, 95% CI: 0.152, 0.992) and Year of study (AOR = 0.389(0.164, 0.922) were significant predictor of level of attitude of Non-Health Science students. Year of study (AOR = 5.159, 95% CI: 1.611, 16.525) was a significant predictor of level of practice of Health Science students.

**Conclusion:**

Significant knowledge difference and attitude difference were observed between students from Arsi University and Adama Science and Technology University.

## Background

The demand for the whole blood transfusion is rising in relation to increased life expectancy, accidents, severe anemia, cancer, chronic diseases, pregnancy-related complication and technological advancements in the healthcare delivery system demanding blood transfusion [1–3].

Among the different types of blood donors, the safest blood comes from voluntary blood donors. In different countries, the largest proportion of hospital obtained blood is replacement donation [4]. The World Health Organization (WHO) policy is to achieve 100% not-paid blood donation practice in 2020 [2].

There is a constant effort made to increase voluntary blood donation practice. Voluntary unpaid donors are the safest group who gives blood regularly [5]. Only 60% of the people in developing countries have adequate knowledge towards blood donation. The blood donation rate in low-income, middle-income, and high-income countries is 3.9, 36.8 and 11.7 per 1000 population, respectively [6, 7].

Willingness to donate blood without expecting financial reward is one major factor to influence blood donation practice. Donor eligibility, negative attitude and lack of education lead to blood shortage in various facilities [8]. Worldwide blood donation practices are increasing day by day, yet it is a big concern for many countries. Safe blood prevents blood borne infections from the donor to the recipient. Furthermore, it saves millions of lives each year. Blood donation is included as the main aspect of the preventive and therapeutic component of the health care delivery system [9]. Blood is a scarce product. There is an imbalance between the demand and supply [2]. In developing countries, the community accessed around 40% of the blood banks supply and from this, 60% are collected from paid blood donation [10]. This is associated with the hesitation to donate blood. People assume that they may develop complication from donating. This is a major misconception underlying the practice [11].

There is a need to establish a global and a nationwide organization in initiating and leading the practice of blood donation. Red Cross and Red Crescent society are institutions working in developing countries to address the blood donation for the mothers delivering, for patients undergoing surgery and for the patients who suffered from accidents [12].

Bleeding might be caused by accidents, medical procedures and giving birth.495,000 women die from bleeding associated with pregnancy and childbirth, which needs early medical interventions [13]. Worldwide people from all age groups require a blood donation to support continuity of life and improve the life quality [14]. This relates to the sophisticated medical surgical procedures requiring blood transfusion [15]. There is a growing need for blood. This is related to the advancement in the healthcare delivery [10]. WHO reported that 38% of voluntarily donated blood comes from those young people aged less than 25. There is a need to motivate young generations to meet 100% voluntary not-paid blood donation [16].

Young students are healthy, active, dynamic, resourceful and receptive; they constitute a greater proportion of the population so that those young students need encouragement and motivation to donate blood voluntarily. Absence of volunteer blood donation is a main challenge seen in our study area. Furthermore, no previous comparative study conducted in this area, this study tried to come up with the following major finding that fills the existing information gap on the level of knowledge, attitude and practice towards voluntary blood donation by comparing Health Science and Non-Health Science students of both universities.

## Methods

### Study area and period

The study was conducted in Adama Science and Technology University (ASTU) and Arsi university regular undergraduate Non-Health Science and Health Science students respectively. ASTU is found in Adama town East Shewa zone in Oromiya regional state. It is 100 km far from the capital city of Ethiopia, Addis Ababa. It has 9737 regular undergraduate students 7205 male & 2512 Female students among them 7448 are 3rd year and above. Arsi University is found in Oromiya regional state, Arsi zone, in Aselatown that is around 170 km far from Addis Ababa. There are around 1130 regular undergraduate Health Science students in this university.764 of them are male and 333 are female students. Among them, 538 are from 3rd year up to 5th year. The study was conducted from April 11–May 2, 2016.

### Study design

An institution based comparative cross-sectional study design was used.

### Population

The Source population was all undergraduate Health Science and Non-Health Science University students and the study population was sampled students who will fulfill the inclusion criteria.

### Inclusion and exclusion criteria

ASTU Non-Health Science and Arsi university Health Science regular undergraduate at least 18 years old and those students who were from third-year up to the fifth-year were included. Those students who were critically ill during the data collection time, distance program students, weekend students, postgraduate students, students who had chronic disease and mentally challenged students were excluded.

### Sample size and sampling procedure

#### Sample size determination

The sample size was calculated using Epi-Info version 21 by considering a 23.6% prevalence of blood donation practice from Ambo study [3], 95% confidence level, 80% power of the study, a risk ratio of 2, and one to one ratio (1:1)in comparison groups. After adding of 5% Non-response rate, the final sample size became 360(in each group it became 180).

#### Sampling procedure

First, the two universities were selected using a stratified sampling method, then the sample was equally distributed between both universities (half of the Health Science students and the other half of Non-Health Science students).Then the sample was proportional allocated using year of study. Finally, the simple random sampling method was used.

#### Operational definition

##### Knowledge

A score of one was given for the correct response and zero for wrong response. Respondents who scored above the mean scored were considered as having good knowledge and others were considered as having poor knowledge.

##### Attitude

A score of one was given for each correct response and zero for the wrong response. Respondents who scored above the mean scored were considered as having favorable attitude and others were considered as having unfavorable attitude.

##### Practice

It was measured by asking about the history of blood donation.

#### Data collection tools

##### Structured questionnaire

The structured questionnaire was adapted after a review of different literatures [3, 11, 13]. The questionnaire had four parts. The first part questions about the socio-demographic characteristics of the respondents. The second, third and fourth part questions about the knowledge, attitude and practice level of blood donation respectively.

##### Data collection procedure

Four data collectors were recruited. One supervisor was used during the data collection period. Training was provided for the data collectors and the supervisor for two days by the principal investigator. The sessions of the training were the objective of the study, the meaning of each question and techniques of interview. In addition, the role of the data collector and the supervisor was covered.

#### Data quality assurance

Training was given to supervisors and data collectors. After completing the training, a pre-test did in rift valley University College Adama campus, accounting 5% of the total sample. During the data collection period, supervisors reviewed for the completeness, consistency, and accuracy of each questionnaire. Corrective measures were taken by discussing with the research team.

#### Data processing and analysis

Data checked manually for completeness and then coded and entered using EpiData version 3.1.The generated data were exported to SPSS version 20. The data were cleaned by visualizing, calculating frequencies and sorting. Frequencies and proportions were computed. The statistical association was done for categorical variables. Significance was determined by using crude and adjusted odds ratios with 95% confidence intervals. To assess the association between the dependent variables and independent variables, bivariable analysis was employed. Then multiple logistic regressions were employed to identify different predictor variables with considering *p*-value less than 0.05. Finally, the results were presented as tables, figures and sentence.

## Result

### Socio-demographic characteristics

In the study, 360 students participated in the study. The median age of Health Science students was 22 with a range of 21–23 and the Non-Health Science students were 23 with a range of 22–24. Among them, 294(81.7%) were males, 233(64.7%) were Orthodox religion followers, 139(38.6%) were Amhara and 199(55.3%) came from urban areas (Table 1).

**Table 1:** Socio-demographic characteristics of Health Science students in Arsi University Health Science students and Adama science and Technology University Non-Health Science students, Ethiopia, April 11–May 2, 2016

Variables		Non-Health Science student		Health Science student		Total (*n* = 360)	
		N	(%)	N	(%)	N	%
Age	18–24	152	84.4	152	84.4	304	84.4
	25–30	28	15.6	28	15.6	56	15.6
Gender	Male	152	84.4	142	78.9	294	81.7
	Female	28	15.6	38	21.1	66	18.3
Religion	Orthodox	117	65	116	64.4	233	64.7
	Protestant	34	18.9	34	18.9	68	18.9
	Muslim	29	16.1	30	16.7	59	16.4
Ethnicity	Amhara	76	42.2	63	35	139	38.6
	Oromo	48	26.7	90	50	138	38.3
	Others	56	31.1	27	15	83	23.1
Original Residence	Rural	74	41.1	87	48.3	161	44.7
	Urban	106	58.9	93	51.7	199	55.3
year of study	Third year	90	50	72	40	162	45
	Fourth Year	50	27.8	65	36.1	115	31.9
	Fifth year	40	22.2	43	23.9	83	23.1

### Level of knowledge of blood donation

The majority of Health Science students 143(79.4%) of them has good knowledge regarding blood donation on the other hand only 25(13.9%) of Non-Health Science students shown to have good knowledge generally there is a significant knowledge difference was observed (Table 2).

**Table 2:** Level of knowledge of blood donation among Health Science students in Arsi University and Non-Health Science students in Adama Science and Technology University, Ethiopia, April 11–May 2, 2016

Variables	Health Science students		Non-Health Science students		χ2	*P* value
	n	%	n	%		
Level of knowledge						
Good	143	79.4	25	13.9	152.779	<0.001^a^
Poor	37	20.6	155	86.1		

^a^Significant association

### Level of attitude on blood donation

Less than half 84(46.7%) and 64(35.6%) of Health and Non-Health Science students have favorable attitudes towards blood donation respectively. Generally, on chi-square test, a significant difference of attitude level observed, Health Science students shown to have a better level of attitude when compared to Non-Health Science (Table 3).

**Table 3:** Level of attitude on blood donation among Health Science students in Arsi University and Non-Health Science students in Adama Science and Technology University, Ethiopia, April 11–May 2, 2016

Variables	Health Science students		Non-Health Science students		χ2	*P* value
	n	%	n	%		
Level of Attitude						
Favorable	84	46.7	64	35.6	4.142	0.042^a^
Non-favorable	96	53.3	116	64.4		

^a^Significant association

### Level of practice of blood donation

Pertaining to blood donation practices 49 (27.2%) and 41 (22.8%) of Health and Non-Health Science students donate blood at least once in their lifetime respectively. A significant difference was observed regarding the level of blood donation practice between health and Non-Health Science students (Table 4).

**Table 4:** Level of practice of blood donation among Health Science students in Arsi University and Non-Health Science students at Adama Science and Technology University, Ethiopia, April 11–May 2, 2016

Variables	Health Science students		Non-Health Science students		χ2	*P* value
	n	%	n	%		
Level of practice						
Yes	49	27.2	41	22.8	0.726	0.394
No	131	72.8	139	77.2		

### Factors associated with the level of knowledge among health science students

Multivariable logistic regression was used to identify factors associated with the level of knowledge about blood donation. Consequently, coefficients were expressed as crude and adjusted OR relative to the referent category. The gender of the students was found as the significant predictor. Accordingly, female Health Science students were 3.2 times more likely to have a better knowledge than male Health Science students were (AOR = 3.150, 95% CI: 1.313, 7.554) (Table 5).

**Table 5:** Factors associated with Level of knowledge on blood donation among Health Science students in Arsi University, Ethiopia, April 11–May 2, 2016

Variables		Knowledge level		COR(95% CI)	AOR(95% CI)
		Good	Poor		
Gender	Male^a^	118	24	1	1
	Female	25	13	2.557(1.148,5.696) ^b^	3.150(1.313,7.554) ^b^
Age	18-24^a^	118	34	1	1
	25–30	25	3	0.416(0.119,1.464)	0.391(0.100,1.528)
Religion	Orthodox^a^	91	25	1	1
	Protestant	27	7	0.944(0.368,2.420)	0.871(0.310,2.449)
	Others	25	5	0.728(0.253,2.096)	0.749(0.242,2.320)
Ethnicity	Amhara^a^	48	15	1	1
	Oromo	74	16	0.692(0.313,1.528)	0.823(0.347,1.951)
	Others	21	6	0.914(0.312,2.683)	0.896(0.270,2.974)
Year of study	Third year^a^	59	13	1	1
	Fourth year	47	18	1.738(0.773,3.906)	2.400(0.993,5.804)
	Fifth year	37	6	0.736(0.257,2.105)	0.890(0.286,2.774)
Residence	Rural^a^	69	18	1	1
	Urban	74	19	0.984(0.477,2.029)	1.027(0.465,2.266)

^a^Reference Category, ^b^Significant association

### Factors associated with level of attitude among health science students

Multivariable logistic regression was used to identify factors associated with attitudes about blood donation. The ethnicity of the students was found as the significant predictor. Health Science students from the Oromo ethnic group were 2.1 times more likely to have a favorable attitude as compared to Health Science students from the Amhara ethnic group (AOR = 2.085, 95% CI: 1.025, 4.243) (Table 6).

**Table 6:** Factors associated with Level of attitude on blood donation among Health Science students in Arsi University, Ethiopia, April 11–May 2, 2016

Variables		Attitude level		COR(95% CI)	AOR(95% CI)
		Favorable	Unfavorable		
Gender	Male^a^	71	71	1	1
	Female	13	25	1.923(0.912,4.057)	1.972(0.884,4.399)
Age	18-24^a^	72	80	1	1
	25–30	12	16	1.200(0.532,2.707)	1.159(0.452,2.967)
Religion	Orthodox^a^	56	60	1	1
	Protestant	18	16	0.830(0.386,1.784)	0.649(0.283,1.493)
	Others	10	20	1.867(0.804,4.332)	1.475(0.607,3.588)
Ethnicity	Amhara^a^	36	27	1	1
	Oromo	36	54	2.000(1.041,3.844) ^b^	2.085(1.025,4.243) ^b^
	Others	12	15	1.667(0.672,4.134)	1.823(0.681,4.884)
Year of study	Third year^a^	32	40	1	1
	Fourth year	31	34	0.877(0.448,1.720)	0.913(0.442,1.886)
	Fifth year	21	22	0.838(0.393,1.787)	0.836(0.356,1.966)
Residence	Rural^a^	43	44	1	1
	Urban	41	52	1.239(0.689,2.229)	1.083(0.576,2.037)
Knowledge	Good^a^	69	74	1	1
	Poor	15	22	1.368(0.657,2.848)	1.346(0.613,2.952)

^a^Reference category, ^b^Significant association

### Factors associated with level of practice among health science students

Multivariable logistic regression was used to identify factors associated with practice of blood donation. The gender of the students was found as the significant predictor. Accordingly, Female Health Science students were 65.7% less likely to donate blood than male Health Science students were (AOR = 0.343, 95% CI: 0.151, 0.779) (Table 7).

**Table 7:** Factors associated with Level of practice of blood donation among Health Science students in Arsi University, Ethiopia, April 11–May 2, 2016

Variables		Practice level		COR(95% CI)	AOR(95% CI)
		Yes	No		
Gender	Male^a^	32	110	1	1
	Female	17	21	0.359(0.170,0.761) ^b^	0.343(0.151,0.779) ^b^
Age	18-24^a^	39	113	1	1
	25–30	10	18	0.621(0.264,1.460)	0.820(0.307,2.188)
Religion	Orthodox^a^	34	82	1	1
	Protestant	8	26	1.348(0.555,3.273)	1.427(0.542,3.762)
	Others	7	23	1.362(0.534,3.473)	1.419(0.522,3.860)
Ethnicity	Amhara^a^	18	45	1	1
	Oromo	24	66	1.100(0.536,2.258)	0.896(0.405,1.982)
	Others	7	20	1.143(0.412,3.168)	1.046(0.338,3.237)
Year of study	Third year^a^	14	58	1	1
	Fourth year	20	45	0.543(0.247,1.192)	0.461(0.195,1.091)
	Fifth year	15	28	0.451(0.191,1.061)	0.466(0.177,1.229)
Residence	Rural^a^	21	66	1	1
	Urban	28	65	0.739(0.381,1.431)	0.792(0.385,1.629)
Knowledge	Good^a^	36	107	1	1
	Poor	13	24	0.621(0.287,1.346)	0.760(0.327,1.765)

^a^Reference category, ^b^Significant association

### Factors associated with level of knowledge among non-health science students

Multivariable logistic regression used to identify factors associated with the level of knowledge about blood donation. Before adjustment, Fifth-year Non-Health Science students were 66.7% less likely to have higher knowledge than third-year Non-Health Science (AOR = 0.333, 95% CI: 0.123, 0.900).However, it was not any more significant after adjustment.

After adjustment, Religion and residence of the students were significant predictors. Accordingly, Protestant religion follower Non-Health Science students were 10.2 times more likely to have a better knowledge than Orthodox religion follower Non- Health Science students (AOR = 10.173, 95% CI: 1.191,86.905). Urban living Non-Health Science students were 71.1% less likely to had good knowledge than rural living Non- Health Science students (AOR = 0.289, 95% CI: 0.094,0.891) (Table 8).

**Table 8:** Factors associated with Level of knowledge on blood donation among Non-Health Science students in Adama Science and Technology University, Ethiopia, April 11–May 2, 2016

Variables		Knowledge		COR(95% CI)	AOR(95% CI)
		Good	Poor		
Gender	Male^a^	22	130	1	1
	Female	3	25	1.410(0.392,5.072)	1.846(0.444,7.674)
Age	18-24^a^	21	131	1	1
	25–30	4	24	0.962(0.303,3.051)	0.861(0.228,3.247)
Religion	Orthodox^a^	20	97	1	1
	Protestant	1	33	6.804(0.879,52.687)	10.173(1.191,86.905)^b^
	Others	4	25	1.289(0.404,4.111)	1.248(0.359,4.331)
Ethnicity	Amhara^a^	10	66	1	1
	Oromo	5	43	1.303(0.417,4.075)	1.171(0.345,3.973)
	Others	10	46	0.697(0.268,1.809)	0.501(0.175,1.435)
Year of study	Third year^a^	9	81	1	1
	Fourth year	6	44	0.815(0.272,2.439)	0.718(0.213,2.421)
	Fifth year	10	30	0.333(0.123,0.900) ^b^	0.341(0.113,1.034)
Residence	Rural^a^	6	68	1	1
	Urban	19	87	0.404(0.153,1.067)	0.289(0.094,0.891) ^b^

^a^Reference category, ^b^Significant association

### Factors associated with the level of attitude among non-health science students

Multivariable logistic regression used to identify factors associated with the level of attitude about blood donation. Before adjustment, other religion follower Non-Health Science students were 3.1 times more likely to have a favorable attitude than orthodox religion follower Non-Health Science students were(AOR = 3.110, 95% CI: 1.108,8.732). Nevertheless, it was not any more significant after adjustment. Gender and year of study of the students were the significant predictor.

Accordingly, Female Non-Health Science students were 61.1% less likely to have a favorable attitude than male Non-Health Science students were (AOR = 0.389, 95% CI: 0.152, 0.992). The fifth year Non-Health Science students were 61.1% less likely to had favorable attitude than third-year Non-Health Science (AOR = 0.389, 95% CI: 0.164, 0.922)(Table 9).

**Table 9:** Factors associated with Level of attitude on blood donation among Non-Health Science students in Adama Science and Technology University, Ethiopia, April 11–May 2, 2016

Variables		Attitude		COR(95% CI)	AOR(95% CI)
		Favorable	Unfavorable		
Gender	Male^a^	50	102	1	1
	Female	14	14	0.490(0.217,1.107)	0.389(0.152,0.992) ^b^
Age	18-24^a^	53	99	1	1
	25–30	11	17	0.827(0.361,1.895)	0.842(0.319,2.225)
Religion	Orthodox^a^	46	71	1	1
	Protestant	13	21	1.047(0.477,2.294)	0.655(0.258,1.663)
	Others	5	24	3.110(1.108,8.732) ^b^	2.778(0.934,8.263)
Ethnicity	Amhara^a^	28	48	1	1
	Oromo	18	30	0.972(0.460,2.053)	0.865(0.367,2.039)
	Others	18	38	1.231(0.594,2.553)	1.731(0.738,4.058)
Year of study	Third year^a^	28	62	1	1
	Fourth year	15	35	1.054(0.497,2.235)	1.036(0.447,2400)
	Fifth year	21	19	0.409(0.190,0.878) ^b^	0.389(0.164,0.922) ^b^
Residence	Rural^a^	25	49	1	1
	Urban	39	67	0.877(0.470,1.634)	0.914(0.448,1.868)
Knowledge level	Good^a^	12	13	1	1
	Poor	52	103	1.828(0.780,4.289	1.883(0.730,4.859)

^a^Reference category, ^b^Significant association

### Factors associated with level of practice among non-health science students

Multivariable logistic regression used to identify factors associated with the level of knowledge. Accordingly, Fourth-year Non-Health Science students were 5.2 times more likely to donate blood than third-year Non- Health Science students (AOR = 5.159, 95% CI: 1.611, 16.525)(Table 10).

**Table 10:** Factors associated with Level of practice of blood donation among Non-Health Science students in Adama Science and Technology University, Ethiopia, April 11–May 2, 2016

Variables		Practice level		COR(95% CI)	AOR(95% CI)
		Yes	No		
Gender	Male^a^	35	117	1	1
	Female	6	22	1.097(0.412,2.918)	1.830(0.619,5.411)
Age	18-24^a^	75	229	1	1
	25–30	15	41	1.428(0.506,4.026)	1.476(0.456,4.782)
Religion	Orthodox^a^	64	169	1	1
	Protestant	12	56	2.586(0.841,7.948)	2.093(0.592,7.397)
	Others	14	45	1.084(0.421,2.792)	1.184(0.422,3.326)
Ethnicity	Amhara^a^	38	101	1	1
	Oromo	32	106	1.786(0.715,4.458)	1.332(0.494,3.591)
	Others	20	63	1.181(0.529,2.638)	0.932(0.375,2.316)
Year of study	Third year^a^	41	121	1	1
	Fourth year	24	91	4.929(1.613,15.056) ^b^	5.159(1.611,16.525) ^b^
	Fifth year	25	58	1.286(0.552,2.996)	1.592(0.620,4.090)
Residence	Rural^a^	37	124	1	1
	Urban	53	146	0.894(0.438,1.822)	0.985(0.430,2.255)
Knowledge level	Good^a^	45	123	1	1
	Poor	45	147	2.162(0.875,5.342)	2.064(0.747,5.701)

^a^Reference category, ^b^Significant association

## Discussion

Maintaining the required level of blood supply is the main concern of various organizations working on health. Therefore, identifying the level of knowledge, attitude and practice is very essential. An attempt was made to identify the knowledge, attitude, practice and factors associated factors with blood donation between Health Science students of Arsi University and Non-Health Science students of Adama Science and Technology University.

The study revealed that 79.4% of Health Science students shown to have good knowledge about blood donation. This is comparable to a study conducted in Addis Ababa university Health Science students, which was (83.6%) [17]; But this is higher when compared with the study conducted on Health Science students in Tamil Nadu, India in which around 35.6% of the respondents shown to have a good level of knowledge [18]. It is also higher than the study conducted on Health Science students of Manipur (9%) [19]. This difference might be related to the background of the students.

The study also revealed that less than half (46.7%) of Health Science students had a favorable attitude. This finding is different from the finding of a study conducted in South Indian in which 87.3% of the respondents show favorable attitude [20]; it is also lower than the study conducted on Addis Ababa University health-science students of Ethiopia, in which 68% of the respondents had a favorable attitude [17]. This difference might occur due to sociocultural difference and educational attributes between the respondents. This suggests the need for more emphasis on blood donation in the Health Science curriculum.

A significant good level of knowledge difference observed between Health Science students (79.4%) and Non-Health Science students (13.9%). In addition, significant favorable attitude differences observed between Health Science students (46.7%) and Non-Health Science students (35.6%). This might be due to the effect of education delivered for Health Science students. In addition, direct exposure in the hospital environment might improve the knowledge and attitude of Health Science students.

Unlike previous reports year of study was not a significant association with knowledge towards blood donation among Health Science students [17, 21]. This might be due to the absence of special education related to blood donation delivered to senior students.

In the study, female Non-Health Science students were 61.1% less likely to have a favorable attitude than male Non-Health Science students did. This might be due to the less frequent mass-media exposure of females. This in turn might decrease access to information provided on blood donation. In addition, it might be due to the cultural taboo. This implies there is a need for health information delivery through campaigns and different sources of information.

Female Health Science students were 65.7% less likely to donate blood than male Health Science students were. This finding is consistent with similar studies [22–24]. This might be related to the limited opportunity and freedom of choice offered for females. This implies that females need to be advised more on the medical benefits of donating blood.

27.2% Health Science students have donated blood. This is contrary to a study conducted in South India in which 10.75% have donated blood [18], in Canada 43.8% have donated blood [25]. This might be due to the difference in the implementation of blood donation campaigns. In addition, the rate of blood donation in developing country is low [26]. This implies the health policy designed in those countries need to give due emphasis to blood donation practice. Furthermore, making Blood Banks available in different health institutions might strengthen the involvement of the institutions in promoting the practice.

Knowledge has no significant association with the level of practice among Health Science students. This is similar to different studies [27, 28]. This might be due to the lack of opportunity and have not asked for donating blood by donor recruitment programs. To increase the number of volunteer blood donors, students need to be constantly encouraged to donate blood through different blood campaigns.

The limitation of this study was the response might have been liable to social desirability bias. The study design cannot assess the cause and effect relationship. In addition, the factors expected to influence knowledge, attitude and practice may not be exhaustive. There could be other factors, which the study did not reveal. It has to be noted that the finding of this study mainly reflects the situation in Adama Science and Technology University and Arsi University. Therefore, the findings should be interpreted with caution.

The result signifies that the universities should establish or res-strengthen blood donation clubs. The students should receive training on voluntary blood donation. Furthermore, both universities need to work with various agencies to remove the misconceptions. Students also need to be motivated with recognitions. The national health policies need to focus on blood donation campaigns through the mobilization of students.

## Conclusion

A significant level of knowledge difference and level of attitude difference observed between the Health Science of Arsi University and Non-Health Science students of Adama science and Technology University. There was no difference in the practice of blood donation between the two groups. The gender of the students was a significant predictor of the level of knowledge and level of practice of Health Science students. The ethnicity of the students was a significant predictor of the level of an attitude of Health Science students. Religion and residence of the students’ was the significant predictor of the level of knowledge of Non-Health Science students. Gender and year of study of the students were significant predictors of the level of an attitude of Non-Health Science students. The year of a study of the students was a significant predictor of the level of practice of Non-Health Science students.

## Abbreviations

AOR: Adjusted Odds Ration
ASTU: Adama Science and Technology University
CI: Confidence Interval
COR: Crude Odds Ration
WHO: World Health Organization
